# Hypoxic Conditions Induce a Cancer-Like Phenotype in Human Breast Epithelial Cells

**DOI:** 10.1371/journal.pone.0046543

**Published:** 2012-09-28

**Authors:** Marica Vaapil, Karolina Helczynska, René Villadsen, Ole W. Petersen, Elisabet Johansson, Siv Beckman, Christer Larsson, Sven Påhlman, Annika Jögi

**Affiliations:** 1 Department of Laboratory Medicine, Center for Molecular Pathology, Skåne University Hospital Malmö, Malmö, Sweden; 2 CREATE Health, Lund University, Lund, Sweden; 3 Department of Surgery, Skåne University Hospital, Malmö, Sweden; 4 Department of Cellular and Molecular Medicine, Centre for Biological Disease Analysis, and The Danish Stem Cell Centre, Faculty of Health Sciences, University of Copenhagen, Copenhagen, Denmark; Sanford Burnham Medical Research Institute, United States of America

## Abstract

**Introduction:**

Solid tumors are less oxygenated than their tissue of origin. Low intra-tumor oxygen levels are associated with worse outcome, increased metastatic potential and immature phenotype in breast cancer. We have reported that tumor hypoxia correlates to low differentiation status in breast cancer. Less is known about effects of hypoxia on non-malignant cells. Here we address whether hypoxia influences the differentiation stage of non-malignant breast epithelial cells and potentially have bearing on early stages of tumorigenesis.

**Methods:**

Normal human primary breast epithelial cells and immortalized non-malignant mammary epithelial MCF-10A cells were grown in a three-dimensional overlay culture on laminin-rich extracellular matrix for up to 21 days at normoxic or hypoxic conditions. Acinar morphogenesis and expression of markers of epithelial differentiation and cell polarization were analyzed by immunofluorescence, immunohistochemistry, qPCR and immunoblot.

**Results:**

In large ductal carcinoma *in situ* patient-specimens, we find that epithelial cells with high HIF-1α levels and multiple cell layers away from the vasculature are immature compared to well-oxygenated cells. We show that hypoxic conditions impaired acinar morphogenesis of primary and immortalized breast epithelial cells grown *ex vivo* on laminin-rich matrix. Normoxic cultures formed polarized acini-like spheres with the anticipated distribution of marker proteins associated with mammary epithelial polarization *e.g*. α6-integrin, laminin 5 and Human Milk Fat Globule/MUC1. At hypoxia, cells were not polarized and the sub-cellular distribution pattern of the marker proteins rather resembled that reported *in vivo* in breast cancer. The hypoxic cells remained in a mitotic state, whereas proliferation ceased with acinar morphogenesis at normoxia. We found induced expression of the differentiation repressor ID1 in the undifferentiated hypoxic MCF-10A cell structures. Acinar morphogenesis was associated with global histone deacetylation whereas the hypoxic breast epithelial cells showed sustained global histone acetylation, which is generally associated with active transcription and an undifferentiated proliferative state.

## Introduction

The tissue-oxygen levels vary considerably between and within different organs. Low oxygenation, hypoxia, can occur locally for numerous reasons such as increased cell proliferation, inflammation, fibrosis, and injury. In the breast, benign sclerotic lesions are linked to increased risk of invasive breast cancer and this risk increases with time and lesion size [1], [2]. These sclerotic lesions are poorly oxygenated, a state that most likely increases with duration and size of the lesion. We hypothesize that persistent hypoxia may play a role in malignant transformation in hypoxic tissue-regions. However, the effect of low oxygenation on non-malignant epithelial cells is not well explored.

The influence of hypoxia in solid tumors and on tumor cells has been more thoroughly studied. With increasing tumor-size the ongoing growth of the cell mass gives rise to elevated intra-tumor pressure and insufficient perfusion leading to hypoxia (reviewed in [3]). Hence, tumors in various organs, including the breast, are poorly oxygenated compared to the corresponding normal tissues. Extensive tumor hypoxia correlates with worse patient outcome and treatment failure [4]. Hypoxia induces a large number of biological responses, such as neovascularization and adapted metabolism. The cellular adaptation to oxygen deprivation is mainly guided by the hypoxia inducible transcription factors, HIF-1 and HIF-2. These dimeric factors contain a unique α-subunit (HIF-1α or HIF-2α) and share the β-subunit (ARNT). HIF-1α and HIF-2α are regulated in a similar manner, primarily by a vast increase in protein stability at low oxygen conditions [5]. Direct HIF transcriptional targets include vascular endothelial growth factor (VEGF), BNIP3 that is involved in cell survival, and the OCT4 and BHLHE40 transcription factors, which are associated with differentiation status and tumor progression [6], [7], [8].

Hypoxic cancer cells, including breast cancer cells, acquire a less differentiated phenotype with expression of stem cell markers [8], [9], [10], [11]. In ductal carcinoma *in situ* of the breast (DCIS), hypoxic cells surrounding the necrotic zones are morphologically dedifferentiated by standard clinical histopathological criteria and the hypoxic cells show no tendency to organize in semi-polarized, ductal-like structures [9]. These unorganized cells show high expression of HIF-1α protein and the mammary epithelial stem cell marker cytokeratin 19 (CK19) [12], [13]. In estrogen receptor (ER) positive tumors the ER expression was down regulated in the hypoxic cells [9], most likely as a part of a hypoxia-induced dedifferentiation process [14]. We hypothesize that hypoxia-driven tumor cell dedifferentiation is one mechanism by which DCIS lesions and pre-malignant cells shift to a malignant and invasive tumor phenotype since a low stage of differentiation correlates to poor outcome in breast cancer and other solid tumors. The HIFs might have direct roles in this process and we have shown that high levels of HIF-2α correlate to poor survival and distant metastasis in breast cancer [12] and neuroblastoma [15]. Whether hypoxia and activation of HIFs play an early role during the tumorigenic process is not known.

To investigate the effect of hypoxia on epithelial polarization and cellular differentiation in non-malignant cells at three dimensional (3D) conditions, we chose two models of extra cellular matrix (ECM)-induced acinar morphogenesis; human breast epithelial cells isolated from normal tissue and the well-characterized immortalized epithelial cells, MCF-10A. In normoxic 3D culture these cells form growth-arrested acinar structures of palisade cells with polarized protein and organelle localization lining an evacuated lumen [13], [16]. Here we show that under hypoxic conditions the cells grow as disorganized cell lumps without the outer polarized cell layer or lumen, and the polarized distribution of marker proteins is disrupted. The hypoxic cells retain their proliferative capacity. In agreement with an impaired differentiation, hypoxic MCF-10A cells had an increased *ID1* (*inhibitor of differentiation*) expression and a sustained global histone acetylation. Cellular adaptation to hypoxia has largely been viewed as a change in hypoxia-driven transcription, but here we demonstrate that protein localization, and not merely protein expression levels, is an additional and potentially clinically important level of cellular adaptation to hypoxia.

## Materials and Methods

### Ethics Statement

Normal breast tissue was obtained from Søllerød Privathospital and Københavns Privathospital with the written consent of individuals, approved by the Regional Scientific Ethical Committees for Copenhagen and Frederiksberg (Den Nationale Videnskabsetiske Komite) (KF) (11) 263995. The data were analyzed anonymously and all clinical investigation was conducted according to the principles expressed in the Declaration of Helsinki.

### 3D-cell Culture

All cell culture was performed at 5% CO_2_, 37°C in humidified cell incubators. Primary breast organoids from healthy donors were dissected from tissue and processed to a single cell suspension. Luminal epithelial cells were sorted in a FACSAria (BD Biosciences) using anti-MUC1 monoclonal antibody (Biogenesis clone 115D8) as described [13]. The cells were grown in overlay cultures on a solidified layer of growth factor reduced ECM-derived substrate (Matrigel, BD, NJ) in DMEM/F12 (Invitrogen) containing 250 ng/ml insulin, 10 µg/ml transferrin, 2.6 ng/ml sodium selenite, 0.1 nM estradiol, 1.4 µM hydrocortisone, 5 µg/ml prolactin, 10 ng/ml EGF and 5% growth factor reduced ECM-derived substrate. Breast epithelial cells from four individuals were separately cultured and analyzed, one sample did not grow in culture. The non-malignant mammary epithelial cells, MCF-10A (a kind gift from Professor J.S. Brugge, Harvard Medical School, Boston, [16], [17]), were kept in culture for no more than seven passages to ensure cell authenticity and maintenance of cell morphology. The MCF-10A 3D-cultures were grown according to the overlay method as previously described [16]. Briefly, the cells were seeded onto a solidified layer of growth factor reduced ECM-derived substrate and grown in DMEM/F12 (Invitrogen) containing 2% horse serum, 0.5 µg/ml hydrocortisone, 100 ng/ml cholera toxin, 10 µg/ml insulin, 5 ng/ml EGF, and 2% growth factor reduced ECM-derived substrate. All cells were cultured in parallel at normoxia (21% O_2_) and hypoxia (1% O_2_, Hypoxystation, Don Whitney, UK) for up to 21 days.

### Immunofluorescence, Immunohistochemistry and Confocal Microscopy

The cultures were fixed, permeabilised, and immunofluorescence stained [16]. The primary antibodies used were: anti-α6-integrin, anti-acetylated-histone H4, anti-laminin-5 (Millipore, MA), anti-Human Milk Fat Globule (HMFG)/MUC1 (Millipore, MA (MCF-10A), Abcam (primary cells)), anti-E-cadherin (Alexis), and anti-Ki-67 (Dako, Denmark). Alexa Fluor-488- or FITC- coupled secondary antibody (Molecular Probes, Invitrogen) was used. Actin was stained with Alexa Fluor-546- or 488-coupled phalloidin (Molecular Probes, Invitrogen), and cell nuclei with 4,6-diamino-2-phenylindole (DAPI, Vector lab). *In situ* cell death was detected with TMR red (Roche, Germany). Confocal images were captured with a Zeiss LSM 710 or Bio-Rad Radiance 2000 confocal system using a 40x oil objective. All confocal images were captured at the z-level with the widest circumference of the acini-like structures. For Ki-67 and cell death calculation at least 200 MCF-10A organoid-cells per experiment and oxygen concentration in three independent experiments were analyzed. For calculation of ID1 positivity 25-165 MCF-10A organoid-cells per experiment and oxygen condition were evaluated in three independent experiments. Cytosolic versus basal protein expression was analyzed by calculating the fraction of mean intracellular (not including membrane structures or the nucleus) to mean basal immunofluorescence signal intensity within the same cell (Fig. S1). Ten cells of different organoids per sample and experiment were analyzed. Statistical analysis was performed with Student’s t-test. Anti-HIF-1α (Millipore, MA), anti-HIF-2α (Novus Biologicals, CO), and anti-ID1 (Millipore, Clone 7D4.2) IHC were performed on PFA-fixed and paraffin-embedded cultures.

### Quantification of 3D-cultures

MCF-10A cells were cultured on ECM-derived substrate in 35 mm plates for 21 days in 21% and 1% O_2_ as described above. Consecutive organoids along the diameter of the plate in 21-day 3D-cultures stained with phalloidin and DAPI were examined (Nikon 10x objective). Polarization was defined as ≥50% of the outer cells being organized in a palisade formation. Size of each cell aggregate was calculated as the average of 4 diameters, measured using Volocity 4 software.

### Quantitative Real-time PCR Analyzes and Immunoblotting

Cells were retrieved from the ECM-derived substrate cultures by use of Dispase (BD, NJ), 180 min at 37°C. Hypoxic cultures were dissolved under hypoxic conditions. RNA isolation (RNeasy, Qiagen), cDNA generation (Reverse transcriptase kit, Applied Biosystems) and quantitative real-time PCR (qPCR) (SYBR green PCR master mix, Applied Biosystems) were performed as previously described [18] and relative expression levels, compared to three previously evaluated reference genes, *UBC*, *YWHAZ*, and *SDHA* [11], were calculated employing geometric averaging [19]. Primers used are listed in Table S1. Cell lysis and immunoblotting were performed as described [10]. Antibodies against AcH4 (Millipore, MA), E-cadherin (Becton Dickinson), HIF-1α (Millipore, MA), and HIF-2α (Novus Biologicals, CO) were diluted 1∶500–1000. Immunodetection of SDHA (Abcam, UK) or actin (Abcam, UK) was used as loading controls.

## Results

### Loss of Polarization was Seen in Hypoxic Cells in the DCIS Lesions

**Figure 1:**
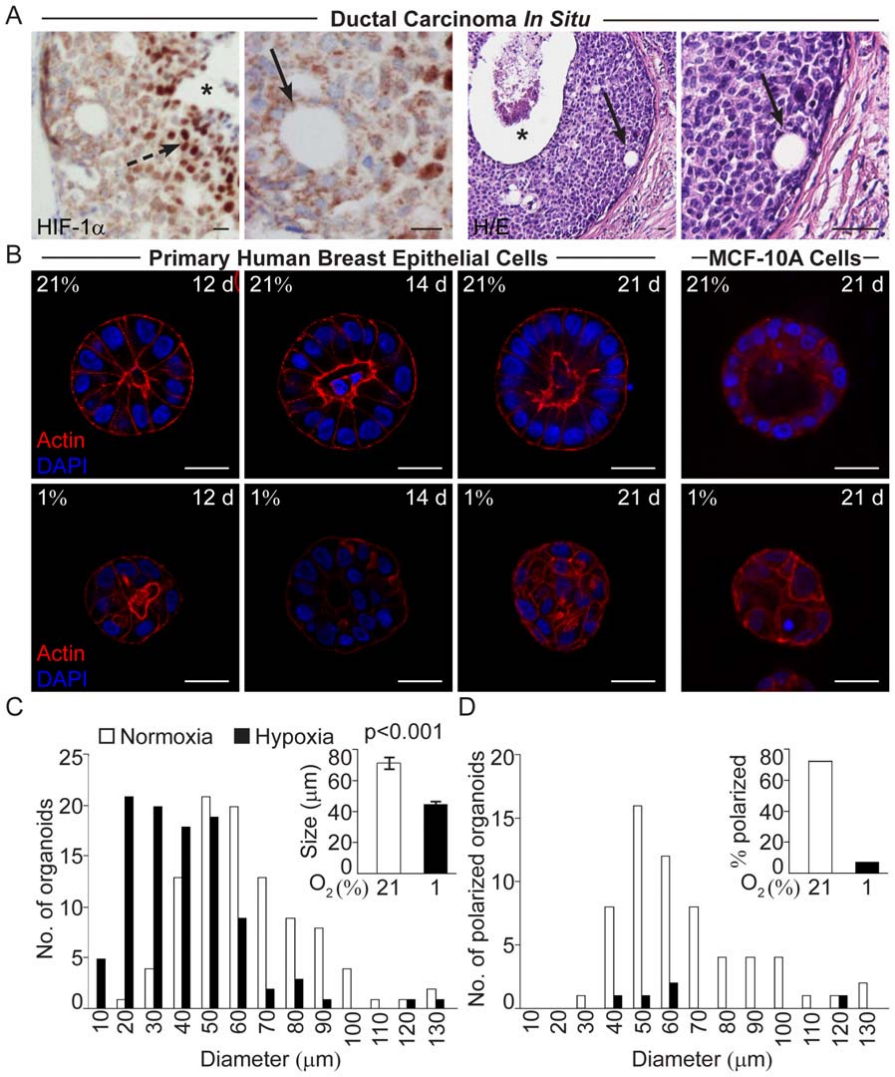
Loss of polarization in hypoxic breast epithelial cells. **A.** Positive HIF-1α IHC staining of hypoxic cells (broken arrow) adjacent to the necrotic zone (star) in ductal carcinoma *in situ* of the breast. Small duct-like formations (arrows) in non-hypoxic regions close to the basal membrane in two different patient specimens of ductal carcinoma *in situ*. H/E; haematoxylin/eosin staining. Size bars 20 µm. **B**. Size and polarization of human breast epithelial cell acini grown on ECM-derived substrate at 21% and 1% oxygen. Actin (phalloidin, red) and nuclear (DAPI, blue) staining of normoxic (upper panel) and hypoxic (lower panel) primary human breast epithelial cells (left panels) and MCF-10A cells (right panel) at the indicated days post-seeding. The primary breast epithelial cell micrographs are from one representative time-series out of three sets of cultured breast cell samples from three different healthy women. All confocal micrographs were acquired at the Z-plane where the depictured acini-like structure had the widest circumference. Size bars 20 µm. **C.** Number of MCF-10A cell organoids of a given diameter (left) and the average size (right) of MCF-10A cell organoids cultured at 21% or 1% oxygen for 21 days on ECM-derived substrate. **D.** Number of polarized MCF-10A cell organoids of the given diameter (left) and the percentage of polarized organoids (right) after 21 days of culture on ECM-derived substrate at 21% or 1% oxygen. Data from one representative experiment out of three is shown. Organoids were considered polarized if 50% or more of the cells in the outer layer formed a palisade.

In DCIS lesions of the *comedo* form, *i.e*. a lesion with several cell layers and a central necrotic zone, the inner cell layers adjacent to the necrosis are hypoxic as demonstrated by HIF-1α staining (Fig. 1A). Closer to the basal membrane intra-lesional ductal-like structures with polarized cells could frequently be found (Fig. 1A). These structures were rarely seen in the cells of the hypoxic zone and we therefore asked whether the lack of these structures is an effect of the hypoxic conditions. To address this question we cultured normal human breast epithelial cells in 3D-cultures at normoxia and hypoxia.

### Hypoxic Human Breast Epithelial Cells form Small and Unorganized 3D-cell Structures

Human breast epithelial cells isolated and enriched from breast tissues [13] from four healthy women were in independent experiments seeded sparsely on top of ECM-derived substrate and cultured at normoxic (21% O_2_) or hypoxic (1% O_2_) conditions for up to 21 days. At normoxia the human primary breast epithelial cells from three of the four women formed acini-like structures of polarized cells with a palisade structure surrounding an evacuated lumen (Fig. 1B). Cells from the fourth woman did not grow in culture. The primary cells of the three breast samples grown in parallel cultures at hypoxia formed non-organized and non-polarized organoids without lumen, lacking resemblance to differentiated mammary acini (Fig. 1B). The immortalized non-tumorigenic MCF-10A cells also form acini-like structures in 3D-cultures on ECM-derived substrate at normoxia (Fig. 1B) [16], while parallel hypoxic cultures formed unorganized structures without polarization (Fig. 1B, D). When measured 21 days post-seeding, the hypoxic structures were significantly (p<0.001) smaller than their normoxic counterparts (Fig. 1B, C). Comparing normoxic and hypoxic structures of the same size (40–60 µm in diameter), revealed a substantial difference in number of organized polarized structures, *i.e.* this feature was not directly associated with the size of the acini-like structures (Fig. 1D). Presence of a polarized palisade cell layer could not be determined in organoids with less than 8 cells in the mid confocal z-plane, therefore these cell clusters were excluded when calculating the fraction of polarized acini (Fig. 1D). The number of such small organoids was higher in hypoxic cultures (Fig. 1C).

### Hypoxic Mammary Epithelial Cells Remained Proliferative Whereas the Normoxic Cells Ceased to Proliferate in Conjunction with Acinar Morphogenesis

**Figure 2:**
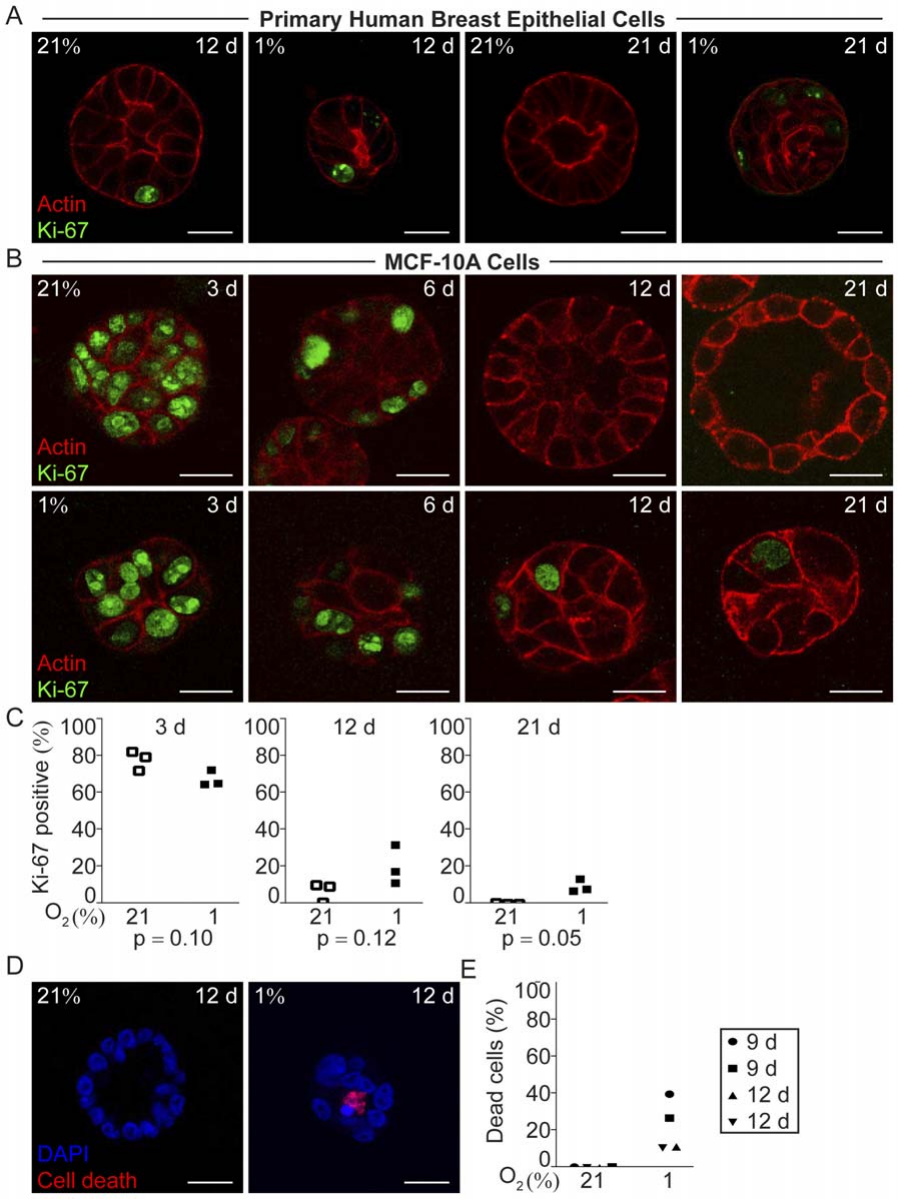
Proliferation and cell death in hypoxic and normoxic 3D-cultures in ECM-derived substrate. A Ki-67 immunofluorescence (green) and actin (red) staining of primary human breast epithelial cells in 3D-culture in ECM-derived substrate at 21% and 1% oxygen for 12, and 21 days. Representative images from one of three independent experiments with breast epithelial cells isolated from different healthy individuals are shown. Size bars 20 µm. **B.** MCF-10A cells stained for Ki-67 (green) and actin (red) after 3, 6, 12, and 21 days of 3D-culture in ECM-derived substrate under normoxic (21%) or hypoxic (1%) conditions. Representative images from one of three independent experiments are shown. Size bars 20 µm. **C.** Percentage of cells with Ki-67 positive nuclei in normoxic and hypoxic MCF-10A cell organoids 3, 12, and 21 days post-seeding, in three independent experiments. Statistical analysis was performed with Student’s paired t-test (p). In each experiment at least 200 cells were included in the calculation. **D.** Cell death in MCF-10A cells grown in 3D-culture under normoxic and hypoxic conditions for 12 days, by *in situ* cell death detection (red), nuclear staining with DAPI (blue). All confocal micrographs were acquired at the Z-plane where the depictured acini-like structure had the widest circumference. Size bars 20 µm. **E.** Percentage of cells with nuclei positive for *in situ* cell death detection in normoxic (21%) and hypoxic (1%) 3D-cultures at 9 and 12 days post-seeding. Data from four experiments are shown. In each experiment at least 200 cells were included in the calculation.

Addressing the question why the hypoxic structures were smaller, we analyzed proliferation by means of Ki-67 expression. At early time points after seeding, the percentage of Ki-67 positive cells was high in both normoxic and hypoxic organoids, as shown in MCF-10A cell 3D-cultures three days post-seeding (Fig. 2B and C). At normoxia, the percentage of Ki-67-expressing MCF-10A cells decreased as acinar morphogenesis took place (Fig. 2B, and C). Also in the forming primary breast epithelial acini the proliferation was low and at day 21 Ki-67 positive cells were virtually absent from the normoxic acini (Fig. 2A). The internal positive control cells growing as monolayer on occasional ECM-derived substrate-free patches were still Ki-67 positive in both normoxic and hypoxic cultures at late time points (Fig. S2). In contrast, the breast epithelial cell organoids formed under hypoxia contained a fraction of Ki-67 positive cells throughout the culture period, albeit the proportion of Ki-67 positive cells decreased with time (Fig. 2A, B, and C). Cell nuclei with mitotic bodies were seen in the hypoxic cells at all studied time points (data not shown). The sustained proliferation in the hypoxic organoids suggests that these cells do not enter the post-mitotic state required for differentiation.

### Higher Incidence of Cell Death was Detected Under Hypoxic Conditions

Since the smaller size of the hypoxic structures could not be attributed to less proliferation we investigated the frequency of cell death. In normoxic MCF-10A cell organoids very few or no dead cells were detected at the investigated time points, 9, 12 and 21 days post seeding (Fig. 2D, E and data not shown). In contrast, we found a higher frequency of cells positive for *in situ* cell death detection in the hypoxic MCF-10A cell structures at all investigated time points (Fig. 2D, E and data not shown), explaining the smaller cell structures formed at hypoxia despite ongoing proliferation.

### Hypoxia Impaired Epithelial Organization of Mammary Epithelial Acini

To further characterize the evident differences in polarization based on morphology in the normoxic and hypoxic cell organoids, we investigated the distribution patterns of three marker proteins associated with mammary epithelial polarization, Alpha6-integrin, laminin 5 and the Human Milk Fat Globule (HMFG/MUC1). Alpha6-integrin is essential for the polarization state in breast epithelium [20]. The integral basal membrane protein laminin 5 is pivotal for the maintenance of epithelial polarization via its contact with the cell-adhesion apparatus [21]. The membrane-bound glycoprotein HMFG/MUC1 is a principal marker of mammary epithelial cell differentiation and polarization [22], [23] that *in vivo* normally accumulates at the apical surface of breast luminal epithelial cells. In breast cancer cells it is expressed in an aberrantly glycosylated form [24], and an increased cytoplasmic fraction have been associated with higher tumor grade in DCIS [25], [26].

**Figure 3:**
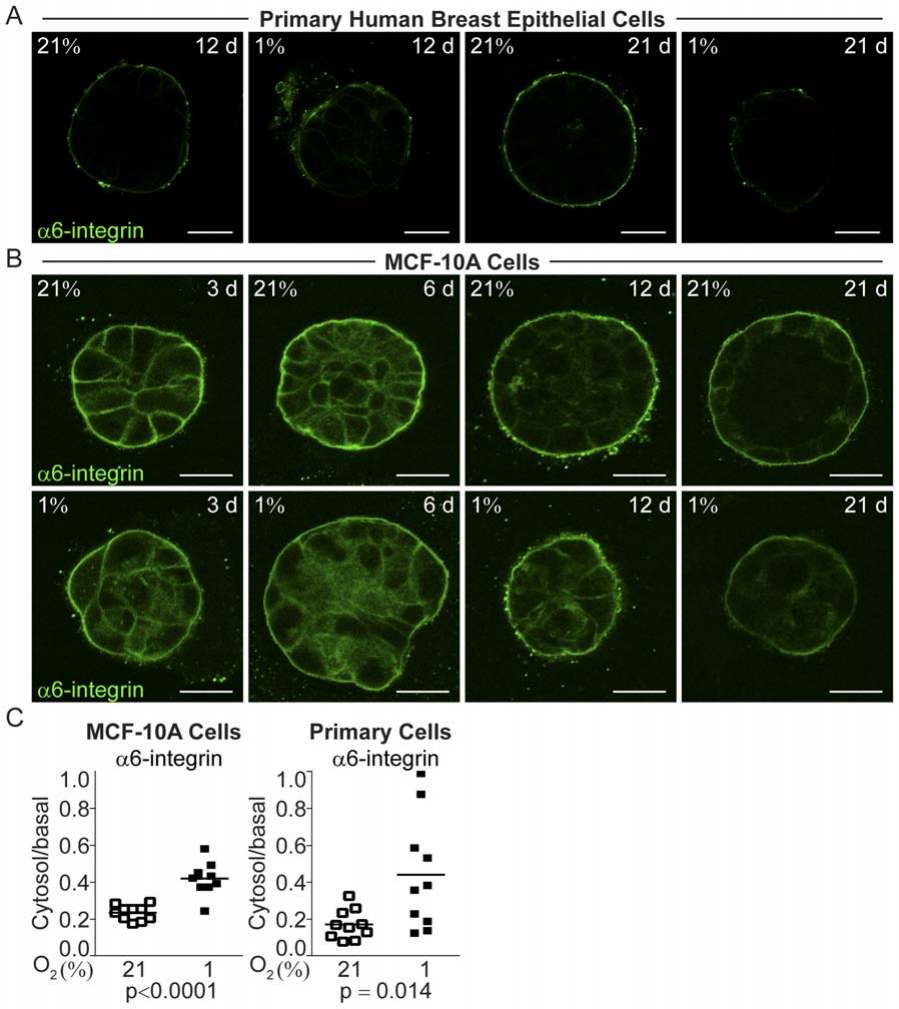
Functional and structural polarization of the human breast epithelial acini-like structures cultured at 21% and 1% oxygen on ECM-derived substrate illustrated by the marker of breast epithelial polarization, α6-integrin. A. Immunofluorescence staining of the polarization marker α6-integrin (green) after 12 and 21 days of culture of primary human breast epithelial cells on ECM-derived substrate under normoxic (21%) or hypoxic (1%) conditions. Images from one representative of three independent experiments with cells from different women are shown. Size bars 20 µm. **B.** α6-integrin (green) staining of normoxic (21%) and hypoxic (1%) MCF-10A cells in 3D-culture at 3, 6, 12, and 21 days post-seeding on ECM-derived substrate. All confocal micrographs were acquired at the Z-plane where the depictured acini-like structure had the widest circumference. Size bars 20 µm. **C.** The ratio of intra cellular to basal cell membrane mean fluorophore intensity in normoxic (open) and hypoxic (black) MCF-10A cell- (left panel) and primary human breast epithelial cell organoids (right panel) at 21 days post-seeding, measured in one representative cell in ten different acini-like structures. Statistical analysis was performed with Student’s t-test (p).

**Figure 4:**
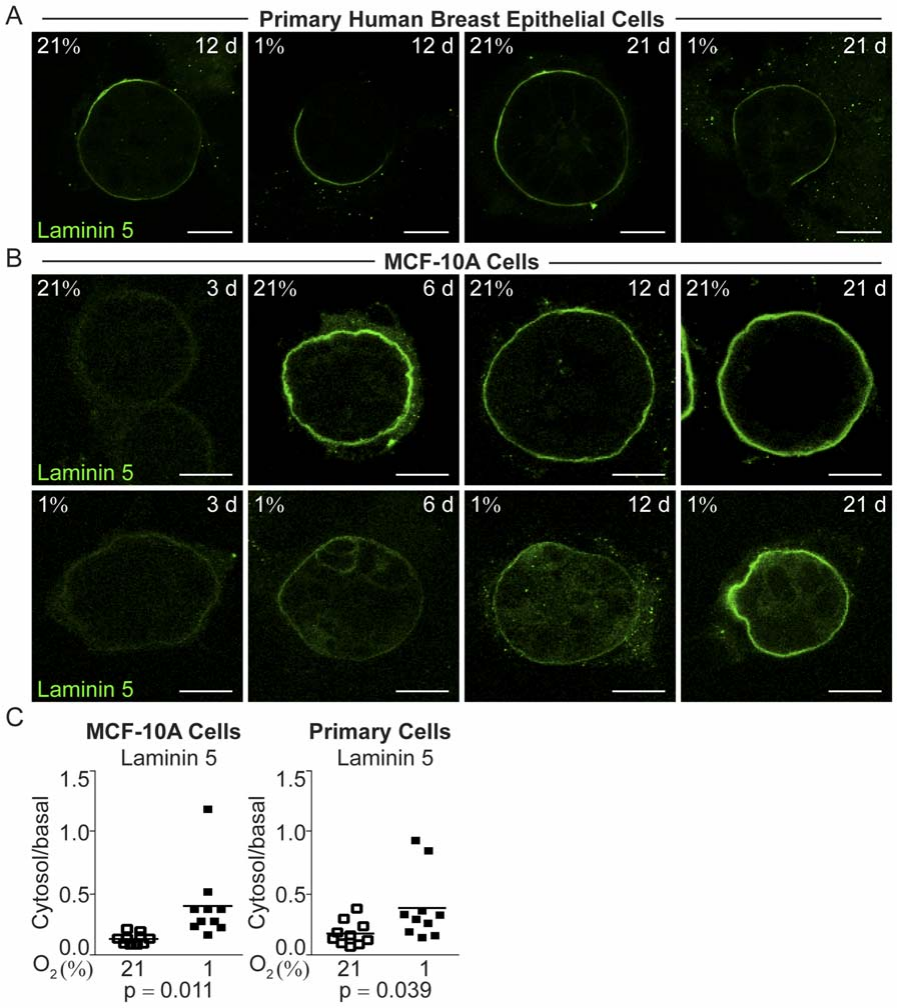
Functional and structural polarization of human breast epithelial cell organoids grown at 21% and 1% oxygen illustrated by the marker of breast epithelial differentiation and polarization laminin 5. A. Laminin 5 (green) immunofluorescence staining of human primary breast epithelial cells after 12 and 21 days of 3D-culture on ECM-derived substrate under normoxic (21%) or hypoxic (1%) conditions. Images from one representative of three independent experiments with cells from different individuals are shown. Size bars 20 µm. **B.** Immunofluorescence of laminin 5 (green) performed after 3, 6, 12, and 21 days of culture of MCF-10 cells in 3D-culture on ECM-derived substrate under normoxic (21%) or hypoxic (1%) conditions. All confocal micrographs were acquired at the Z-plane where the depictured acini-like structure had the widest circumference. Size bars 20 µm. **C.** The ratio of intra cellular to basal cell membrane mean fluorophore intensity in normoxic (open) and hypoxic (black) MCF-10A cells (left panel) and primary human breast epithelial cells (right panel) 21 days post-seeding, measured in one representative cell in ten different organoids. Statistical analysis was performed with Student’s t-test (p).In agreement with the non-malignant status of both the primary breast epithelial and the MCF-10A cells we found HMFG/MUC1 to have a polarized localization in normoxic acinar cells 21 days post-seeding (Fig. 5A, B). The basal, as opposed to apical, localization of this protein is in agreement with MCF-10A cells showing little apical polarization as previously reported [27]. HMFG/MUC1 displayed a decrease in polarized localization in both primary breast epithelial and MCF-10A cells at hypoxia (Fig. 5A, B). In the MCF-10A cells there was a significant difference in the ratio of intra-cellular to basal localization at hypoxia compared to normoxia (p<0.0001, Fig. 5C). The ratio of cytosolic to basal membrane localization could not be reliably determined in the primary breast epithelial cells. The *MUC1* mRNA expression was significantly decreased in the hypoxic MCF-10A cells after 21 days of 3D-culture (p = 0.013, Fig. 5D).

**Figure 5:**
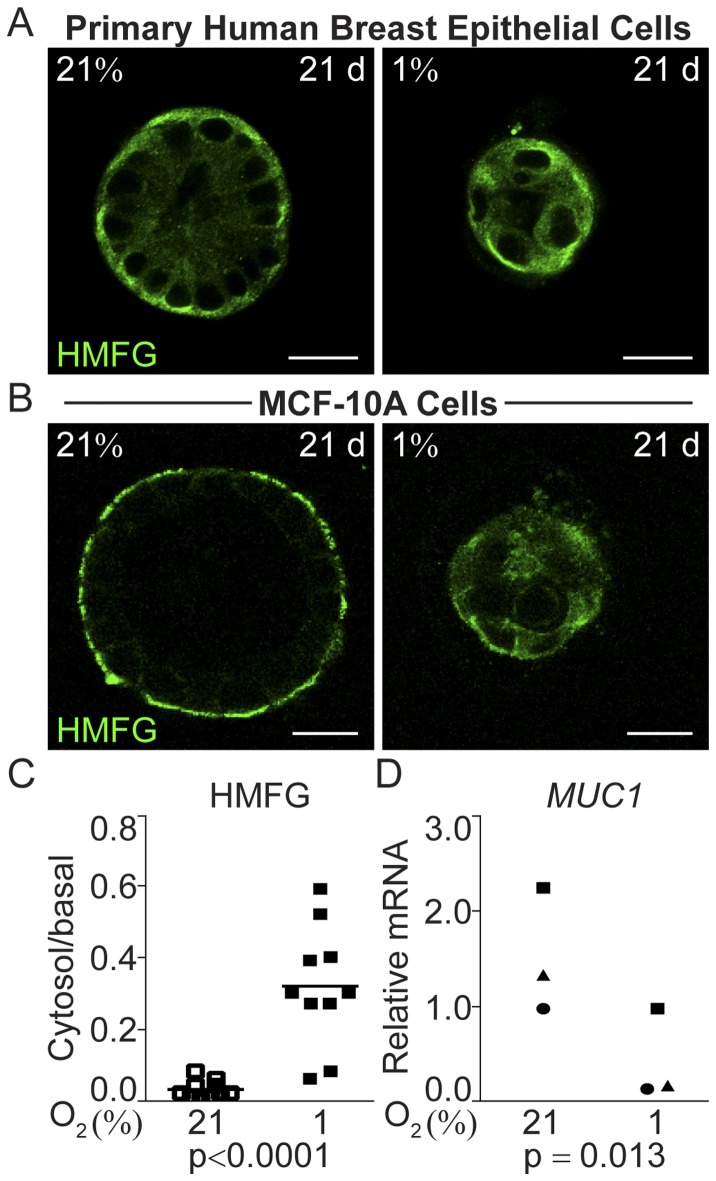
Localization of Human Milk Fat Globule (HMFG) in human breast epithelial cells at normoxia and hypoxia. **A.** Cellular localization of HMFG (green) in primary human breast epithelial cells after 21 days of 3D-culture on ECM-derived substrate at normoxic (21%) or hypoxic (1%) conditions. Images from one representative of three independent experiments with cells from different individuals are shown. Size bars 20 µm. **B.** Staining of HMFG (green) on 3D-cultures of MCF-10A cells after 21 days of culture on ECM-derived substrate at normoxic (21%) or hypoxic (1%) conditions. All confocal micrographs were acquired at the Z-plane where the depictured acini-like structure had the widest circumference. Size bars 20 µm. **C.** The ratio of intra cellular to basal cell membrane mean fluorophore intensity in normoxic (open) and hypoxic (black) MCF-10A cell organoids at 21 days post-seeding, measured in one representative cell in ten different acini-like structures. Statistical analysis was performed with Student’s t-test (p). **D.** Relative mRNA levels of *MUC1* in normoxic (21%) and hypoxic (1%) 3D-cultures of MCF-10A cells 21 days post-seeding, showing data from three independent experiments. Statistical analysis was performed with Student’s paired t-test (p).

All of these mammary epithelial markers showed a significant loss of polarized localization under hypoxic culture conditions (Fig. 3, 4, and 5). In normoxic primary breast epithelial cell cultures, α6-integrin localized to the basolateral surface of the entire acini-structures (Fig. 3A). At hypoxia, this uniform localization was disrupted (Fig. 3A). The ratio of cytoplasmic to basal expression was significantly increased in the breast epithelial cells of hypoxic organoids 21 days post-seeding (p = 0.014, Fig. 3C). In MCF-10A, the polarized rim-cells had basolateral α6-integrin localization (Fig. 3B), while the hypoxic MCF-10A cell organoids displayed a reduced and non-polarized expression of α6-integrin, with significantly increased ratio of intra-cellular to basal expression compared to normoxic cells (p<0.0001, Fig. 3B and C).

Laminin 5 was barely detectable in MCF-10A cells 3 days post-seeding in both normoxia and hypoxia (Fig. 4B). As the normoxic MCF-10A cells differentiated into acini the basal accumulation of laminin 5 increased (Fig. 4B). At hypoxia, intra-cellular localization of laminin 5 was evident at all time-points studied (Fig. 4B), although weak at day 3 post-seeding. The ratio of intra-cellular to basal membrane localization was significantly increased in hypoxic compared to normoxic MCF-10A organoids (p = 0.011, Fig. 4C). In the primary human breast epithelial cells laminin 5 was generally more difficult to detect (Fig 4A, B), but quantitative analysis of the ratio of cytosolic to basal membrane localization revealed a significant increase in cytosolic localization, i.e. decreased polarization (p = 0.039, Fig. 4C).

### Epithelial-to-mesenchymal-transition could not be Detected in the Hypoxic MCF-10A cell 3D-cultures

Our data suggests that hypoxia inhibits polarization and differentiation of non-malignant cells in the acinar morphogenesis model. Epithelial-to-mesenchymal-transformation (EMT) was reported to occur in hypoxic tumors [28], [29]. To test whether the hypoxia-impaired differentiation was associated with EMT E-cadherin with decreased expression as a hallmark of EMT [30], was analyzed. E-cadherin was present in cell membranes at cell-cell contact surfaces of both normoxic and hypoxic MCF-10A cell 3D-structures (Fig. S3A). *E-cadherin* mRNA levels increased at hypoxia compared to normoxia 21 days post-seeding and E-cadherin protein levels increased with time in 3D-culture both in normoxia and hypoxia (Fig. S3B and C). Also, *Vimentin* expression increased in hypoxic cells at 21 days post-seeding (Fig. **S**3B). As loss of E-cadherin and increase in vimentin are expected features of EMT we conclude that a hypoxia-driven EMT of the MCF-10A cells did not occur. However EMT is a process associated with cancer invasion and MCF-10A cells do not grow in an invasive manner [17].

### Hypoxia-induced Gene Expression

**Figure 6:**
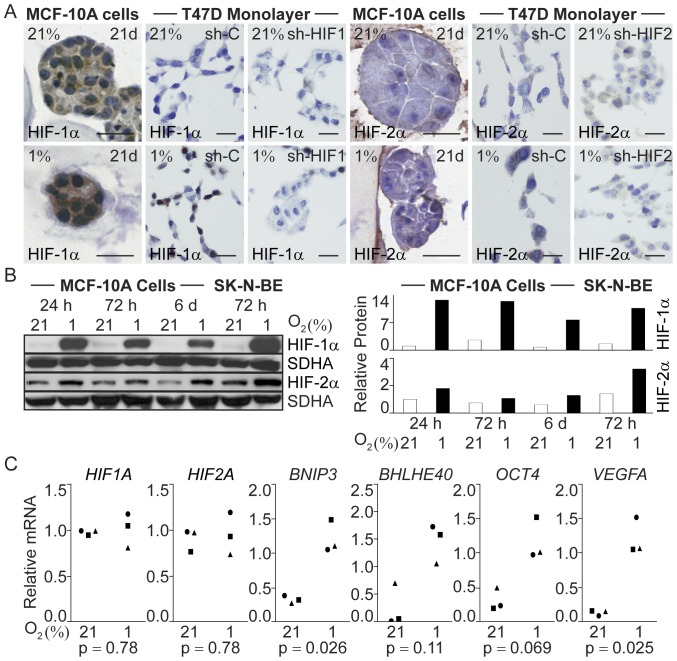
Expression of HIF-1α, HIF-2α, and HIF-target genes in normoxic and hypoxic breast epithelial cells. A. HIF-1α and HIF-2α immunohistochemical staining of MCF-10A acini-like structures after 21 days of 3D-culture at normoxia (21%) or hypoxia (1%). Sh-RNA-treated T47D breast cancer cells grown as monolayer and exposed to normoxia or hypoxia for 24h were used as controls. All cells were fixed in PFA and paraffin-embedded. Size bars 20 µm. **B.** Immunoblot analysis (left panel) of HIF-1α and HIF-2α in protein extracts of MCF-10A cells cultured in monolayer at 21% and 1% oxygen for the indicated period of time. Normoxic and hypoxic SK-N-BE cell extracts were used as controls. Quantification of the HIF signal intensity relative to the loading control (SDHA) (right panel). **C.** Relative mRNA expression of *HIF1A*, *HIF2A*, and the HIF-target genes *BNIP3*, *BHLHE40*, *OCT4*, and *VEGFA* in normoxic and hypoxic MCF-10A cells retrieved from 3D-cultures 21 days post-seeding. Data are from three independent experiments and statistical analysis was performed with Student’s paired t-test (p).

Addressing the mechanism(s) behind the impaired differentiation of human breast epithelial cells in hypoxic 3D-cultures, we analyzed the activity and accumulation of the two primary transcriptional regulators of cellular adaptation to oxygen deprivation, HIF-1α and HIF-2α. HIF protein levels in response to prolonged hypoxia are not well studied in any cellular system, but our previous data suggest that the relative importance of HIF-2α may increase with time [12], [15]. Some degree of increased accumulation in HIF-1α and HIF-2α protein could be detected in paraffin-embedded hypoxic MCF-10A organoids 21 days post-seeding (Fig. 6A). Hypoxic accumulation of both proteins was detected in cell extracts of MCF-10A cells grown as monolayer for up to six days (Fig. 6B). The relative mRNA levels of both HIF-1α and HIF-2α were similar in normoxic and hypoxic 3D-cultures after 21 days (Fig. 6C) in agreement with the primary regulation of these proteins being at the level of protein stabilization. Under normoxic conditions HIF-1α and HIF-2α become ubiquitinated and degraded, a process specifically inhibited at hypoxia leading to protein accumulation. Upon reoxygenation the HIFs are again targeted for degradation and have a half-life of a few minutes [5]. Therefore, the time-consuming process of protein recovery from the ECM-derived substrate cultures has not allowed us to detect HIF-1α and HIF-2α protein levels in the 3D-cultures. Instead, we tested if HIF-induced transcription occurred after 21 days of hypoxia by analyzing the mRNA levels of a panel of established HIF-target genes. We found increased expression of *BNIP3*, *BHLHE40, OCT4*, and *VEGFA* (Fig. 6C) in the hypoxic structures 21 days post-seeding, suggesting that one or both of the HIFs are transcriptionally active in the MCF-10A cell 3D-cultures at 21 days of hypoxia.

### Hypoxia Induced Expression of the Negative Regulator of Mammary Epithelial Differentiation ID1

**Figure 7:**
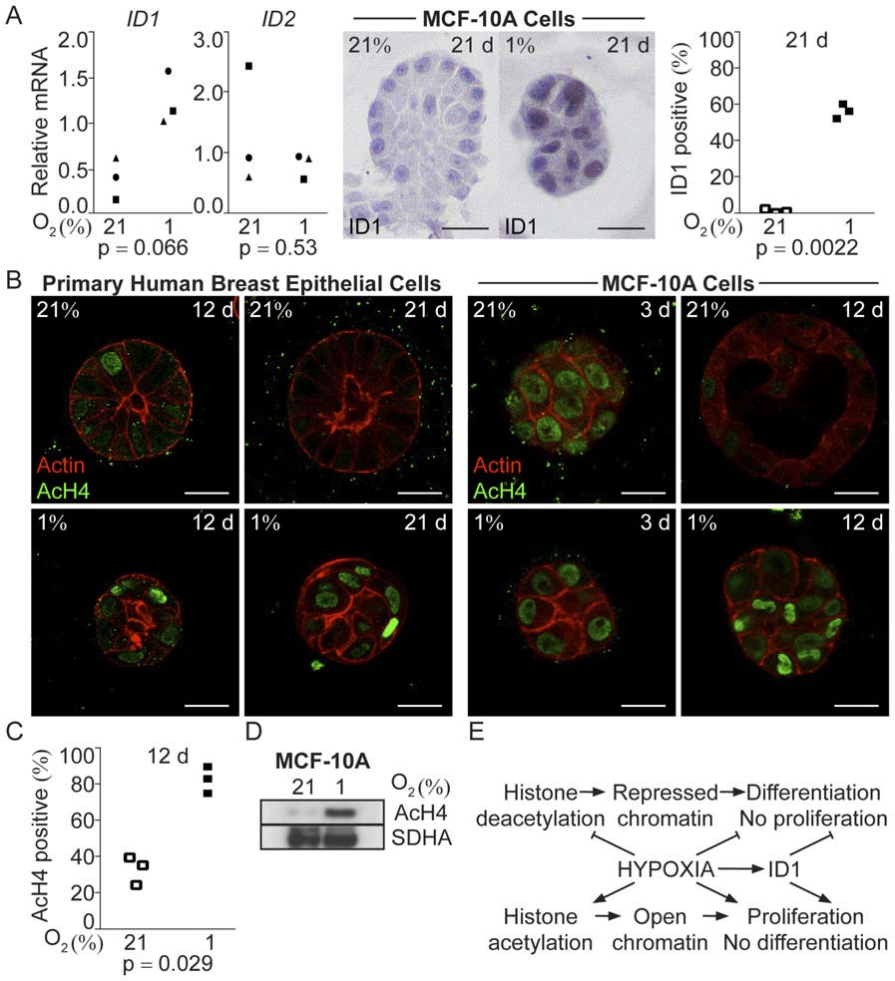
ID1 expression and histone acetylation in breast epithelial cells in normoxic compared to hypoxic 3D-cultures. **A.** Relative mRNA expression of the transcription modulating ID factors, *ID1* and *ID2* in MCF-10A cells after 21 days of 3D-culture on ECM-derived substrate under normoxic and hypoxic conditions (left). Showing data from three independent experiments. Statistical analysis was performed with Student’s paired t-test (p). Immunohistochemical staining for ID1 on paraffin-embedded MCF-10A acini-like structures after 21 days of 3D-culture at normoxia (21%) or hypoxia (1%) (right). Size bars 20 µm. **B.** Acetylated histone 4 (AcH4) visualized by immunofluorescence (green) in normoxic (21%) and hypoxic (1%) human primary breast epithelial cell (left) and MCF-10A cell (right) acini-like structures at the indicated days post seeding. Actin was visualized by phalloidin staining (red). Size bars 20 µm. **C**. Percentage of MCF-10A cells in acini-like structures with global histone acetylation, i.e. positive for AcH4, 21 days post-seeding at normoxia (open boxes) and hypoxia (black boxes), showing data from three independent experiments. At least 200 cells were calculated in each experiment. Statistical analysis was performed by Student’s paired t-test (p). **D.** Immunoblot of AcH4 in MCF-10A cells in 3D-culture at 21% (left) and 1% (right) on ECM-derived substrate for 10 days. SDHA was used as a loading control. **E.** Non-malignant breast epithelial cells grown on differentiation-inducing ECM have an organized cell shape and a high degree of deacetylated histones; these cells differentiate and become post-mitotic. Contrary, breast epithelial cells grown as monolayer without ECM do not receive/accept signals to induce differentiation, leading to sustained global histone acetylation and opening of the chromatin for transcription, resulting in impaired differentiation and/or dedifferentiation accompanied with cell proliferation. Hypoxia, including hypoxic induction of ID1, promotes a proliferative and undifferentiated state in breast epithelial cells despite contact with the ECM.

ID transcription modulating factors are regulated by hypoxia [10], [18], [31]. The ID proteins negatively regulate the activity of a number of tissue-specific basic helix-loop-helix transcription factors instrumental during development and differentiation of numerous organs. In mammary gland differentiation, forced expression of ID1 impairs differentiation and abolishes milk production. ID2 is necessary for full mammary gland differentiation and lactation (reviewed in [32]). After 21 days of 3D-culture, the hypoxic MCF-10A cell organoids had increased ID1 and unchanged ID2 mRNA expression compared to their normoxic counterparts (Fig. 7A). Immunohistochemical detection of ID1 in paraffin-embedded MCF-10A cell organoids 21 days post-seeding showed distinct nuclear staining in the hypoxic cells, whereas the cells of the normoxic organoids had very little ID1 (Fig. 7A). A statistically significant increase in the percentage of ID1-positive nuclei was seen in hypoxic MCF-10A organoid cells at 21 days post-seeding compared to their normoxic counterparts (p = 0.0022, Fig. 7A right panel), consistent with the observed impaired differentiation at hypoxia.

### Sustained Global Histone Acetylation in Hypoxic Human Breast Epithelial Cells in 3D-culture

Acinar morphogenesis is associated with global histone deacetylation and chemical inhibition of histone deacetylation blocks differentiation and formation of organized acinar structures in response to ECM [33]. We therefore hypothesized that the lack of differentiation and organization of mammary epithelial cells in hypoxia might be mediated by loss of histone deacetylation, i.e. the chromatin structure remains open favoring proliferation and low stage of differentiation. The nuclei of cells in hypoxic 3D-cultures of both primary breast epithelial cells and MCF-10A cells stained strongly positive for acetylated histone H4 (AcH4) at all time points studied, whereas the normoxic cells lost global histone 4 acetylation with acini formation (21d resp. 12d) (Fig. 7B). The proportion of cells strongly positive for AcH4 was significantly higher in hypoxic compared to normoxic MCF-10A organoid cells, exemplified at 12 days post-seeding (p = 0.029, Fig. 7C). Moreover, immunoblotting showed increased levels of AcH4 in extracts of cells from hypoxic MCF-10A cell 3D-cultures 10 days post-seeding (Fig. 7D). Histone deacetylation in 3D-cultures is associated with chromatin compaction and decreased nuclear diameter [33]. We found that hypoxic MCF-10A cells had significantly larger nuclear diameter (p<0.05, n = 24 (21%) and n = 30 (1%), in average 14% larger).

To test if the histone acetylation status merely reflects cycling cells we compared parallel cultures stained for Ki-67 and AcH4. While virtually all the hypoxic cells were AcH4 positive (Fig. 7C), only approximately 20% of the cells were Ki-67 positive (Fig. 2C). These data were corroborated by double staining experiments (data not shown). We conclude that although acetylation of H4 may be necessary for cell proliferation it appears not to impose cell cycle progression on it own under the studied conditions.

## Discussion

The epithelium serves as a selective permeability barrier, a function made possible by epithelial cell polarity. Cellular polarization is a feature of differentiation guided by positional cues from components of the ECM, as well as adjacent cells [21], [34]. Loss of epithelial polarity is a sign of low differentiation and a hallmark of malignancy [20]. The 3D-culture assays in ECM-derived substrate enable studies of the processes of breast epithelial polarization and differentiation at *in vivo*-like conditions [16].

Hypoxia has profound effects on tumor cell behavior *in vitro* as well as on cancer prognosis. We have reported that hypoxia leads to a less differentiated cell phenotype in breast cancer and that high HIF-2α expression associates with unfavorable outcome and metastasis [9], [12]. Here we use breast epithelial cell cultures on laminin-rich ECM-derived substrate to study the potential impact of hypoxia on acinar morphogenesis and normal breast epithelial development. Acinar morphogenesis is the result of numerous processes affecting cell shape, cytoskeletal and nuclear matrix organization, chromatin state, and gene expression [35], [36]. We found that hypoxia impairs ECM-induced acinar morphogenesis by affecting several of these processes. Notably, hypoxia led to sustained cell proliferation and as the transition into a post-mitotic state is an explicit hallmark of terminal differentiation, we conclude that hypoxia impairs cellular differentiation of non-malignant human mammary epithelial cells.

Morphology and the sustained proliferative capacity suggested that hypoxia impairs polarization and organization of mammary epithelial cells, a conclusion supported by the aberrant localization and expression of the mammary epithelial differentiation/polarization markers, HMFG/MUC1, laminin 5, and α6-integrin. These marker proteins and their localization also harbor prognostic information in breast cancer. Laminin 5 is normally deposited at the basal surface of acinar cells but in the hypoxic organoid cells shown here, laminin 5 is also present in the cytoplasm, similar to the localization in invasive breast cancer [37]. The monoclonal antibody used here recognizes the γ2-chain, which is unique for laminin 5. High expression and intra-cellular localization of the γ2-chain were reported in dedifferentiated budding tumor cells of colorectal cancer and found to correlate with poor outcome and incidence of distant metastasis [38], [39]. In breast cancer, decreased expression of HMFG/MUC1 is associated with low overall patient survival, low stage of tumor cell differentiation, and increased incidence of distant metastasis [26], [40]. Our findings that the hypoxic organoids have reduced expression and deviant localization of HMFG/MUC1, further strengthens the conclusion that the hypoxic mammary epithelial cells adopt an immature and cancer-like phenotype.

In an attempt to address the importance of the HIFs in the observed impairment of mammary epithelial polarization/differentiation, we silenced both HIF-1α and HIF-2α separately and in combination, in MCF-10A cells by use of viral transduction with shRNA constructs. The procedure led to loss of the ability to form polarized acini also at normoxia (data not shown). This result was also seen with the control viral shRNA-constructs, hence the results were not due to HIF specific effects.

The breast epithelial cells cultured at hypoxia maintained a high degree of global histone acetylation through out the 21-day experiment, whereas the chromatin of the normoxic cells became deacetylated with progression of acinar morphogenesis. In developing rat brain, abrogation of deacetylation impaired development and delayed expression of differentiation markers [41]. Thus, the finding that hypoxic cells had sustained global histone acetylation provides a putative mechanism for the hypoxic inhibition of epithelial cell differentiation and acinar morphogenesis. ECM-induced acini formation is linked to cell shape-dependent global histone deacetylation, whereas conventional monolayer culture results in general histone acetylation favoring transcriptional activity, proliferation and a low state of differentiation [33]. Our data suggest that mammary epithelial cells in hypoxic organoids phenotypically mimic cells in 2D culture lacking contact with the differentiation-inducing ECM (Fig. 7E). Future efforts should be directed towards investigating the effects of hypoxia on histone acetyl transferases and deacetylases, and their association to epithelial polarization and differentiation.

We report here that hypoxia leads to changed expression levels of genes influential in cell differentiation, i.e. *OCT4* and *ID1*, in breast epithelial cells in 3D-culture. The OCT4 homeo-domain transcription factor is associated with self-renewal and stemness, and is a HIF-2 target gene [42]. Sustained expression of *OCT4* in embryonic stem cells prevents differentiation [42], [43]. Thus, the observed increase of *OCT4* expression in hypoxic MCF-10A acini could be a direct HIF-2 effect, promoting an undifferentiated phenotype. The primary mode of action of the ID proteins is by sequestering the ubiquitous partners of the tissue-specific differentiation-regulating bHLH transcription factors [44], though they can also bind directly to the tissue-specific bHLH factors themselves [45]. Our finding that *ID1* expression increased in the undifferentiated hypoxic MCF-10A structures is in agreement with the previous observations that overexpression of ID1 suppresses mammary epithelial differentiation [32], [46]. *ID1* silencing induced differentiation and quiescence in mammary epithelial cells and ECM-induced differentiation is associated with ID1 down regulation [47]. In addition, ectopic ID1 expression in mammary epithelial cells induces both proliferation and apoptosis [48] similar to what we find here in the hypoxic structures. ID1 expression also harbor prognostic information in breast cancer as ID1 expression increase with tumor grade and is an independent prognostic marker [49], [50]. Furthermore, ID1 has been suggested to induce CyclinD1 expression [32]. Taken together, ID1-driven inhibition of differentiation is a plausible mechanism for the impaired acinar morphogenesis accompanied by cancer-like expression of marker genes at hypoxia. ID2, on the other hand, is reported to be necessary for full mammary epithelial differentiation and is expressed in the mammary gland late during pregnancy [48] and *ID2* expression was not induced in our hypoxic 3D-cultures corroborating their undifferentiated status.

As stabilization and activation of the HIF transcription factors are major mechanisms behind cellular adaptation to hypoxia, changes in gene transcription have been in focus in models explaining the adaptation process. However, the hypoxia-induced changes in protein localization within multi-cellular structures reported here add an additional level of regulation at which reduced oxygen pressure can affect cell differentiation and potentially tumor progression. Clearly, this level of regulation may have clinical impact since the differentiation marker proteins, and their localization within the cells, studied here carry prognostic information in breast cancer.

Our present findings suggest that hypoxia traps normal breast epithelial cells in an undifferentiated, proliferative state, which if occurring *in vivo* would increase the risk for tumor-initiating genetic aberrations to become manifest in a proliferating population of cells. Although the scenario we envisage is a situation of local hypoxia due to over-proliferation leading to high local oxygen consumption that is not instantly compensated for by *de novo* vascularization, there are indeed indications that overall anemic situations can be linked to higher cancer incidences. Populations living at high altitudes in the Andes have increased frequency of paraganglioma [51] and congenital heart disease with cyanosis in infants is associated with increased occurrence of neuroblastoma [52].

Tissue hypoxia is a phenomenon that usually occurs locally and according to our view, such a situation would create a time-window at which immature, progenitor-like cells exist and proliferate due to the hypoxic environment and thus could be prone to genetic hits of genes not expressed at the differentiated stage. Especially breast tissue, with its reiterating cycles of cell growth, differentiation and cell death over decades in each individual would be the tissue of choice to expect that local overgrowth could occur, possibly hormone driven. In addition, in the clinical setting, benign sclerotic breast lesions are associated to increased risk of invasive breast cancer and the risk increases with time and lesion size [1], [2]. As these sclerotic lesions are poorly oxygenated, hypoxia may play a role in the malignant transformation in such lesions and possibly other zones with low oxygenation for alternate reasons (e.g. inflammation, poor perfusion). We suggest that these hypoxic effects on epithelial cell differentiation can contribute to tumorigenesis in addition to previously described mechanisms showing hypoxia-induced stromal contributions to tumor initiation and progression [53].

### Conclusions

We show here that hypoxia impairs ECM-induced differentiation and acinar morphogenesis of non-malignant primary human mammary epithelial cells as well as the immortalized MCF-10A cells. Despite contact with laminin-rich ECM the hypoxic mammary epithelial cells maintained a non-differentiated phenotype resembling cells cultured in absence of ECM-components i.e. they were proliferative and could not form organized 3D-structures (Fig. 7E). Loss of polarization and loss of differentiated epithelial structures combined with proliferation are inherent features of breast cancer. The impaired differentiation and polarization in hypoxic 3D-cultured cells was associated with increased expression of the transcriptional modulator ID1, known to counteract mammary epithelial differentiation *in vivo* and *in vitro*. Furthermore, the global deacetylation that takes place with progression of acinar morphogenesis in normoxic cultures did not occur at hypoxia. The cancer-like phenotype of the hypoxic mammary epithelial cells and disorganized 3D-growth lead us to suggest that hypoxia may play a role already at stages of tumor initiation.

## Supporting Information

Figure S1 **Example of location of intracellular (left) and basal (right) areas used for measuring mean immunofluorescence signal.**
(TIF)

Figure S2 **Ki-67 (green) expression in MCF-10A cells growing in monolayer within the 3D-cultures 21 days post-seeding.** Visualization of actin was by phalloidin (red). Size bar 40 µm.
(TIF)

Figure S3 **Epithelial-to-mesenchymal-transition could not be detected in the hypoxic MCF-10A cells in 3D-cultures.** **A.** Immunofluorescence staining of E-cadherin after 21 days of 3D-culture on ECM-derived substrate at normoxic (21%) and hypoxic (1%) conditions. The confocal micrographs were acquired at the Z-plane where the depictured acini-like structure had the widest circumference. Size bars 20 µm. **B.** Relative mRNA expression levels of *E-cadherin* and *Vimentin* in normoxic and hypoxic 3D-cultures after 21 days showing data from three independent experiments. Statistical analysis was performed with Student’s paired t-test (p). **C**. E-cadherin protein levels in MCF-10A cells recovered from normoxic (21%) and hypoxic (1%) 3D-cultures after 3, 12, and 21 days, analyzed by immunoblot.
(TIF)

Table S1 **Sequences of the QPCR primers.**
(PDF)
