# Supplementary figures and images for: Hypoxic Conditions Induce a Cancer-Like Phenotype in Human Breast Epithelial Cells
Source: PLoS One. 2012 Sep 28;7(9):e46543. doi: 10.1371/journal.pone.0046543 (PMC3460905; doi:10.1371/journal.pone.0046543)

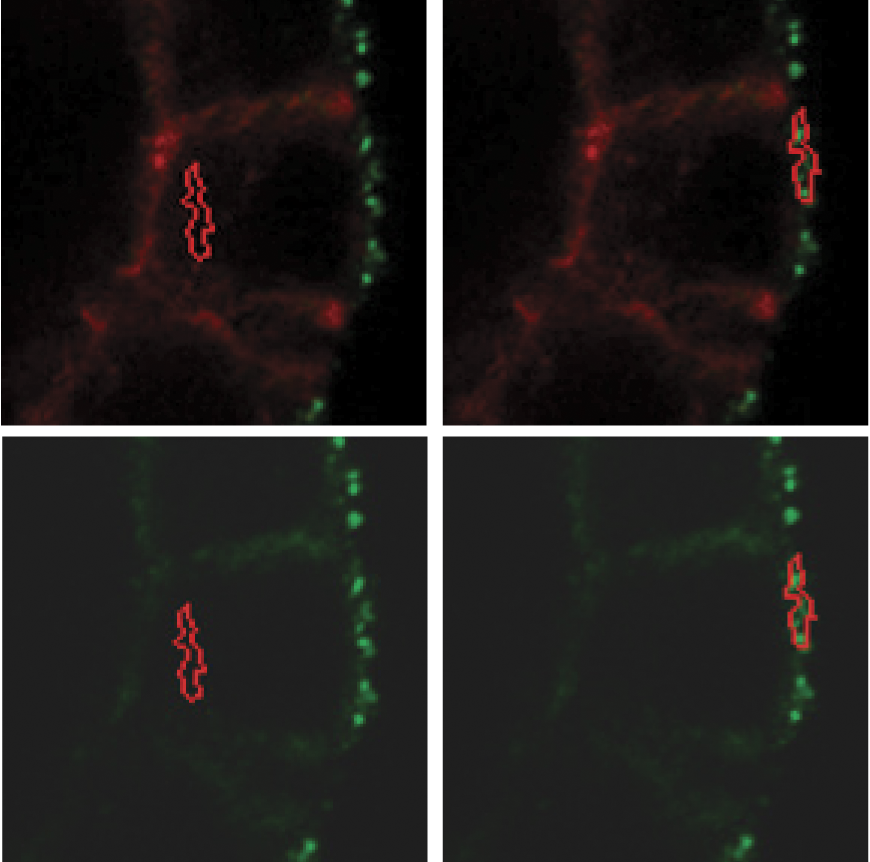

Supplement: Figure S1 — Example of location of intracellular (left) and basal (right) areas used for measuring mean immunofluorescence signal. (TIF) [file pone.0046543.s001.tif]

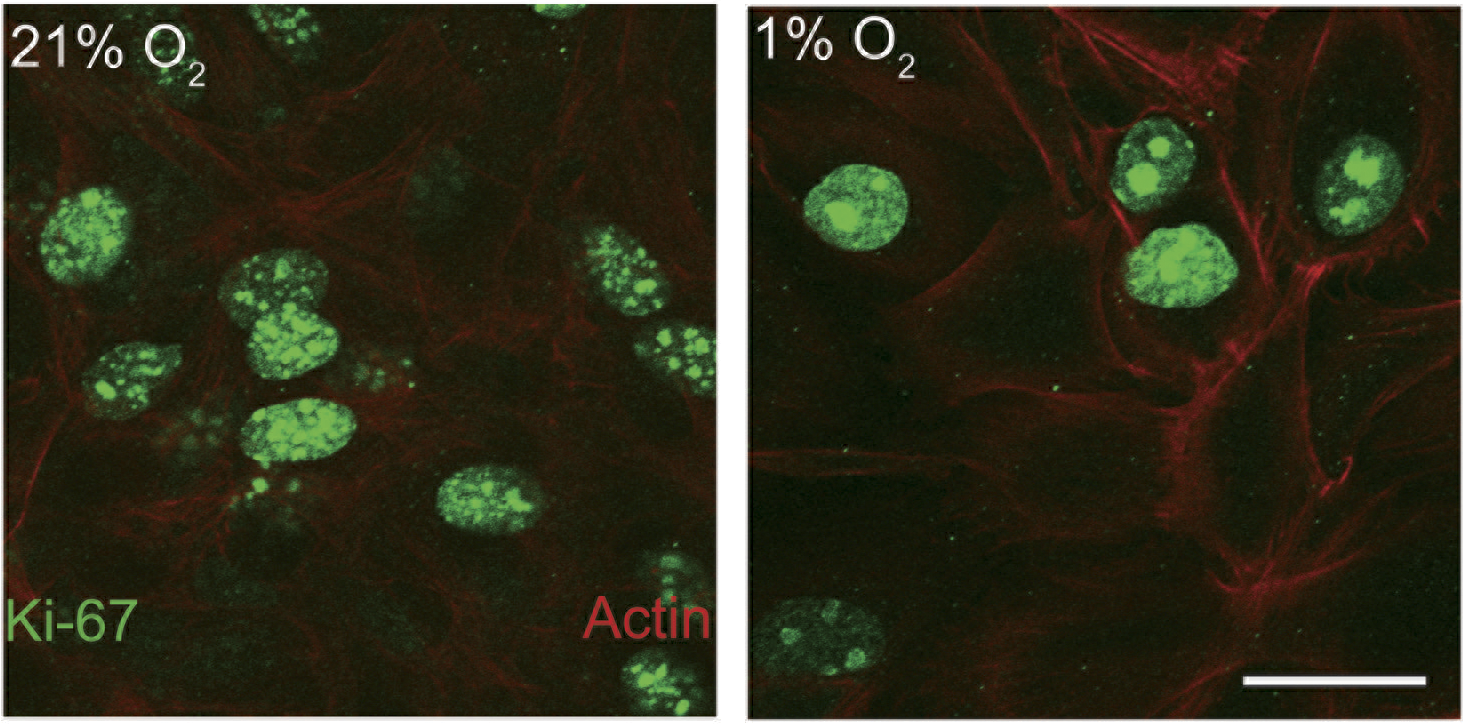

Supplement: Figure S2 — Ki-67 (green) expression in MCF-10A cells growing in monolayer within the 3D-cultures 21 days post-seeding. Visualization of actin was by phalloidin (red). Size bar 40 µm. (TIF) [file pone.0046543.s002.tif]

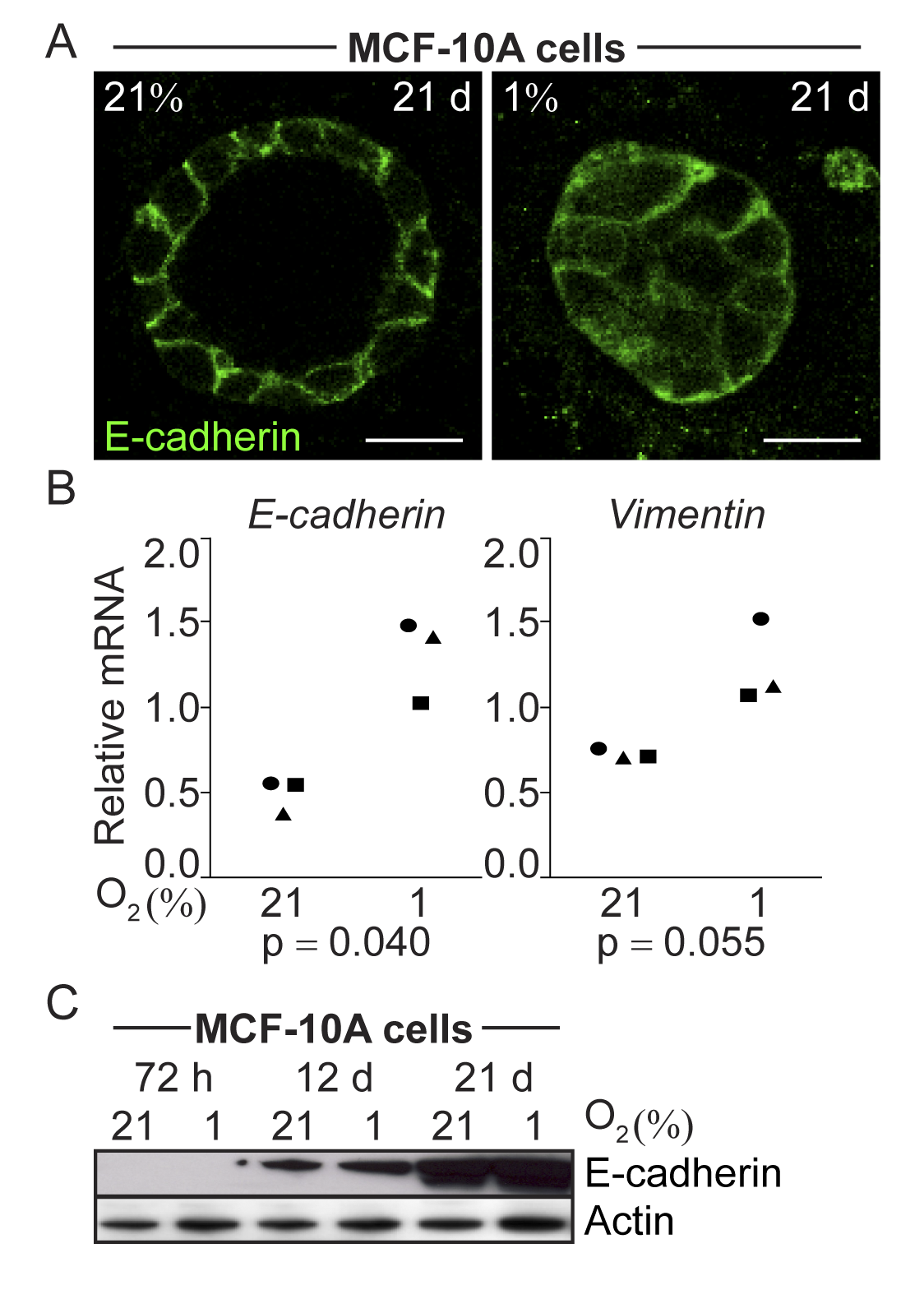

Supplement: Figure S3 — Epithelial-to-mesenchymal-transition could not be detected in the hypoxic MCF-10A cells in 3D-cultures. A. Immunofluorescence staining of E-cadherin after 21 days of 3D-culture on ECM-derived substrate at normoxic (21%) and hypoxic (1%) conditions. The confocal micrographs were acquired at the Z-plane where the depictured acini-like structure had the widest circumference. Size bars 20 µm. B. Relative mRNA expression levels of E-cadherin and Vimentin in normoxic and hypoxic 3D-cultures after 21 days showing data from three independent experiments. Statistical analysis was performed with Student’s paired t-test (p). C. E-cadherin protein levels in MCF-10A cells recovered from normoxic (21%) and hypoxic (1%) 3D-cultures after 3, 12, and 21 days, analyzed by immunoblot. (TIF) [file pone.0046543.s003.tif]
