# Supplementary material for: Hypoxic Conditions Induce a Cancer-Like Phenotype in Human Breast Epithelial Cells
Source: PLoS One. 2012 Sep 28;7(9):e46543. doi: 10.1371/journal.pone.0046543 (PMC3460905; doi:10.1371/journal.pone.0046543)
Supplement: Table S1 — Sequences of the QPCR primers. (PDF) [file pone.0046543.s004.pdf]

Supplemental Table 1. Primers for quantitative PCR

Gene	*Forward primer*	*Reverse primer*
UBC	5'-ATTTGGGTCGCGGTTCTTG-3'	5'-TGCCTTGACATTCTCGATGGT-3'
YWHAZ	5'-ACTTTTGGTACATTGTGTGGCTTCAA-3'	5'-CCGCCAGGACAAACCAGTAT-3'
SDHA	5'-TGGGAACAAGAGGGCATCTG-3'	5'-CCACCACTGCATCAAATTCATG-3'

Gene	*Forward primer*	*Reverse primer*
HIF-1α	5'-TTCCAGTTACGTTCCTTCGATCA-3'	5'-TTTGAGGACTTGCGCTTTCA-3'
HIF-2α	5'-GCTCTCCCACGGCCTGTA-3'	5'-TTGTCACACCTATGGCATATCACC-3'
VEGF	5'-AGGAGGAGGGCAGAATCATCA-3'	5'-CTCGATTGGATGGCAGTAGCT-3'
BNIP3	5'-AAAATATTCCCCCCAAGGAGTTC-3'	5'-ACGCTCGTGTTCCTCATGCT-3'
OCT4	5'-GAGAACCGAGTGAGAGGCAACC-3'	5'-CATAGTCGCTGCTTGATCGCTTG-3'
BHLHE40	5'-CAGTGGCTATGGAGGAGAATCG-3'	5'-GCGTCCGTGGTCACTTTTG-3'
ID1	5'-CTACGACATGAACGGCTGTTA-3'	5'-CTTGCTCACCTTGCGGTTCT-3'
ID2	5'-TCAGCCTGCATCACCAGAGA-3'	5'-CTGCAACAGGATGCTGATA-3'
E-cadherin	5'-TGAGTGTCCCCCGGTATCTTC-3'	5'-CAGTATCAGCCGCTTTCAGATTTT-3'
Vimentin	5'-ACACCCTGCATTCTTTCAGACA-3'	5'-GATTCCACTTTGCGTTCAAGGT-3'
Mucin1	5'-CGCCGAAAGAACTACGGGCAGCTG-3'	5'-CAAGTTGGCAGAAGTGGCTGCCAC-3'
